# Supplementary material for: Impact of Optimized Ku–DNA Binding Inhibitors on the Cellular and In Vivo DNA Damage Response
Source: Cancers (Basel). 2024 Sep 26;16(19):3286. doi: 10.3390/cancers16193286 (PMC11475570; doi:10.3390/cancers16193286)
Supplement: Supplementary file 1 [file cancers-16-03286-s001.zip › cancers-3185404-supplementary.pdf]

Supplementary Data

Impact of Optimized Ku–DNA Binding Inhibitors on the Cellular and In Vivo DNA Damage Response

Pamela L. Mendoza-Munoz [1], Narva Deshwar Kushwaha [2], Dineshsinha Chauhan [2], Karim Ben Ali Gacem [3,4], Joy E. Garret [5] Joseph R. Dynlacht [5], Jean-Baptiste Charbonnier [3], Navnath S. Gavande [2,6,*] and John J. Turchi [1,7,*]

[1] Department of Medicine, Indiana University School of Medicine, Indianapolis, IN 46202, USA

[2] Department of Pharmaceutical Sciences, Eugene Applebaum College of Pharmacy and Health Sciences, Wayne State University, Detroit, MI 48201, USA

[3] Institute for Integrative Biology of the Cell (I2BC), Institute Joliot, CEA, CNRS, Université Paris-Sud, 91198 Gif-sur-Yvette Cedex, France.

[4] Structure-Design-Informatics, Sanofi R&D, Vitry sur Seine 94400, France

[5] Department of Radiation Oncology, Indiana University School of Medicine, Indianapolis, IN 46202, USA

[6] Molecular Therapeutics Program, Barbara Ann Karmanos Cancer Institute, Wayne State University, Detroit, MI 48201, USA.

[7] NERx Biosciences, Indianapolis, IN 46202, USA

* Correspondence: ngavande@wayne.edu (N.S.G.); jturchi@iu.edu (J.J.T.);
Tel.: +1-313-577-1523 (N.S.G.); +1-317-278-1996 (J.J.T.); Fax: +1-313-577-2033 (N.S.G.); +1-317-274-0396 (J.J.T.)

1) Synthetic Experimental Details:

General. All chemicals used for synthesis were purchased from Aldrich, Alfa Aesar, Acros, Fisher Scientific, AK Scientific, and Combi-Blocks Chemical Co. (USA) and used without further purification. Anhydrous solvents were obtained from Fisher Scientific or Aldrich and used directly. All reactions involving air- or moisture-sensitive reagents were performed under a nitrogen atmosphere. ^{1}H NMR spectra were recorded at 400 MHz using Bruker AV NMR spectrometer. ^{13}C NMR spectra were recorded at 101 MHz using Bruker AV NMR spectrometer. ^{13}C Attached-Proton-Test (APT) NMR were recorded at 101 MHz using Bruker AV NMR spectrometer. The signals of CH and CH_3 are negative, but CH_2 and quaternary carbons including the solvent carbon are positive. ^{19}F NMR spectra were recorded at 376 MHz using Bruker AV NMR spectrometer. The chemical shifts were reported as δ ppm relative to TMS, using the residual solvent peak as the reference unless otherwise noted. All coupling constants (*J*) are given in hertz. Data are reported as follows: chemical shift, multiplicity (s = singlet, d = doublet, t = triplet, q = quartet, br = broad, m = multiplet), number of protons, and coupling constants. Thin layer chromatography was performed using Merck silica gel 60 F-254 thin layer plates, which were developed using one of the following techniques: UV fluorescence (254 nm), alkaline potassium permanganate solution (0.5% w/v) or ninhydrin (0.2% w/v) and iodine vapors. The products were purified with column chromatography. LCMS analyses of compounds were obtained using a Shimadzu LC- 20 AT instrument connected to a Shimadzu 1300 HPLC system, and both instruments were connected to a Shimadzu photodiode array (PDA) UV detector. The chemical purity of target compounds was ≥95% determined by analytical HPLC (Shimadzu LC- 20 AD instrument). A C18 reverse phase column (Nova-Pak® 4 μm, 3.9 mm x 1.5 mm) was used as the stationary phase, and water/acetonitrile (both containing 0.1% TFA) was used as the mobile phase (gradient 10−90% ACN/H_2O, flow 1 mL/min, run time 20 min). UV absorbance at wavelengths of 220 and 254 nm were recorded.

All final compounds were purified by recrystallization or flash column chromatography, and the analytical and spectroscopic data confirmed their purity and structures, as detailed in the experimental procedures.

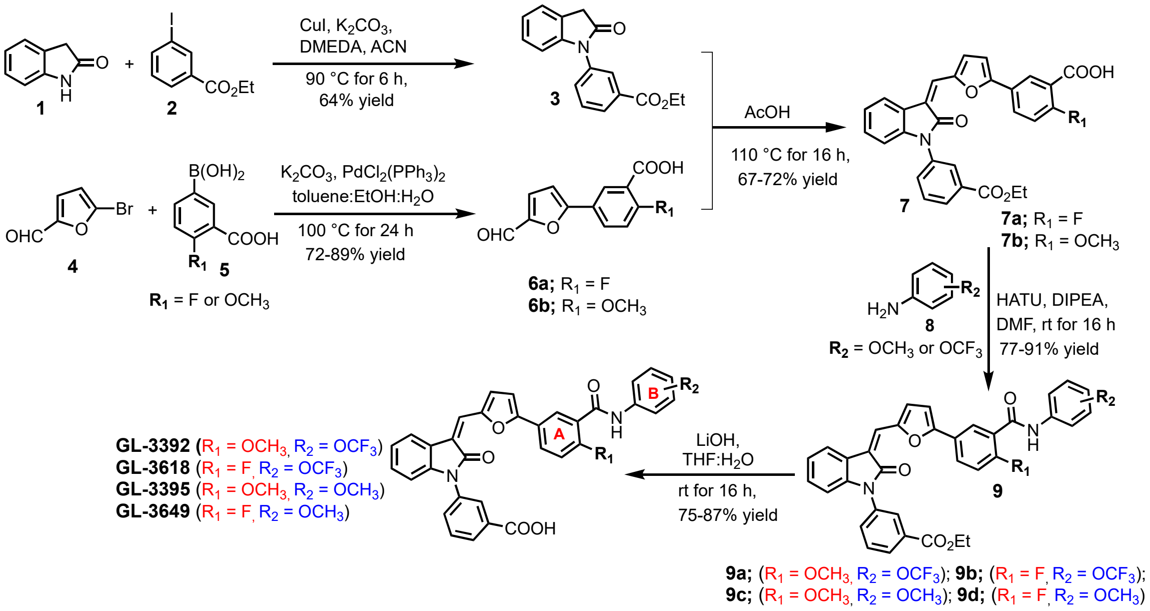

Supplementary **Figure S1.** General synthetic scheme for synthesis of target compounds **GL-3392, GL-3618, GL-3395** and **GL-3649**.

Synthesis of ethyl 3-(2-oxoindolin-1-yl) benzoate (**3**). To the stirred suspension of indolin-2-one (0.5 g, 3.75 mmol, 1 eq.) and ethyl 3-iodobenzoate (1.03 g, 3.75 mmol, 1 eq) in acetonitrile (15 mL) was added K_2CO_3 (1.03 g, 7.51 mmol, 2 eq). The mixture was degassed with argon for 5 min, and then 1,2-dimethylethylenediamine (0.06 mg, 0.75 mmol, 0.2 eq), and CuI (0.071g, 0.375 mmol, 0.1 eq) were added. The resultant mixture was heated at 90 °C for 6 h, then the mixture was cooled to room temperature, filtered through celite, and washed with ethyl acetate (2 × 20 mL). The filtrate was washed with brine solution. The organic layer was dried over Na_2SO_4 and concentrated under reduced pressure. The crude residue was purified by column chromatography using 5-20% EtOAc in hexanes as the eluent to give ethyl 3-(2-oxoindolin-1-yl) benzoate (**3**) as a pale yellow solid (0.675 g, 64% yield). TLC: 50% EtOAc in hexanes, R_f = 0.6; visualized with UV. ^{1}H NMR (400 MHz, DMSO): δ 8.03 – 8.00 (m, 1H), 7.97 (brs, 1H), 7.73 -7.72 (m, 1H), 7.36 (d, J = 7.62 Hz, 1H), 7.21(t, J = 7.62 Hz, 1H), 7.07 (t J = 6.88 Hz, 1H), 6.72 (d, J = 7.37 Hz, 1H) 4.34 (q, J = 7.14 Hz, 2H), 3.77 (s, 2H), 1.32 (t, J = 5.6 Hz, 3H). ^{13}C NMR (101 MHz, DMSO): δ 173.9, 165.0, 144.3, 134.9, 131.4, 131.2, 130.1, 128.4, 127.3, 124.9, 124.7, 122.6, 108.5, 61.1, 35.4, 14.1. MS (ESI) m/z = 282.1 [M + H]$^+$.

General procedure for the synthesis of compounds 6a-6b.

A solution of K_2CO_3 (2 equiv) in water (10 mL) was added to a mixture of 5-bromo-2-furaldehyde (1 eq.) and 5-borono-2-fluorobenzoic acid/5-borono-2-methoxybenzoic acid (1.2 eq) in toluene/ethanol (1:1, v/v, 40 mL). The mixture was degassed with nitrogen for 10 min, and then $Pd(PPh_3)_4$ (0.05 eq.) was added. The reaction mixture was stirred at 100 °C for 24 h, then the reaction mixture was cooled to room temperature, filtered through celite, and washed with water and ethanol (2 × 10 mL). The solvent was evaporated under reduced pressure to give a solid residue. The residue was diluted with water (20 mL) and the pH was adjusted to 2-3 by the addition of 6 N HCl solution, the precipitate was formed. The solid precipitate was filtered and washed with 20−30% EtOAc in hexane to afford the title product.

2-fluoro-5-(5-formylfuran-2-yl)benzoic acid (**6a**). yellow solid (72% yield). TLC: 5% MeOH in DCM, R_f = 0.1; visualized with UV. ^{1}H NMR (400 MHz, DMSO): δ 13.5 (s, 1H), 9.62 (s, 1H), 8.31 – 8.28 (m, 1H), 8.15 – 8.11 (m, 1H), 7.66 (t, J = 3.29 Hz, 1H), 7.51 – 7.45 (m, 1H), 7.38 (t, J = 3.54 Hz, 1H). ^{13}C NMR (101 MHz, DMSO): δ 178.0, 164.5, 162.7, 160.1, 156.2, 151.9, 131.2 – 131.1(d, $J_{C\text{-}F}$ = 14.11 Hz, 1C), 128.1, 125.3, 120.3 – 120.2 (d, $J_{C\text{-}F}$ = 12.25 Hz, 1C), 118.4– 118.2 (d, $J_{C\text{-}F}$ = 22.81 Hz, 1C), 109.3. MS (ESI) m/z = 233.1 [M - H]$^-$.

5-(5-formylfuran-2-yl)-2-methoxybenzoic acid (**6b**). Yellow solid (89% yield). TLC: 5% MeOH in DCM, R_f = 0.1; visualized with UV. ^{1}H NMR (400 MHz, DMSO): δ 12.95 (brs, 1H), 9.57 (s, 1H), 8.10 (s, 1H), 7.99 (d, J = 9.25 Hz, 1H), 7.63 (s, 1H), 7.25 (d, J = 9.25 Hz, 2H), 3.88 (s, 3H). ^{13}C NMR (101 MHz, DMSO): δ 177.5, 166.7, 159.0, 157.6, 151.4, 129.7, 127.3, 125.8, 122.1, 120.8, 113.3, 107.9, 56.1. MS (ESI) m/z = 245.1 [M - H]$^-$.

General procedure for the synthesis of compounds 7a-7b.

Ethyl 3-(2-oxoindolin-1-yl) benzoate **3** (1 eq.) and 2-fluoro-5-(5-formylfuran-2-yl)benzoic acid (**6a**)/5-(5-formylfuran-2-yl)-2-methoxybenzoic acid (**6b**) (1 eq.) were dissolved in glacial acetic acid (20 mL). The reaction mixture was refluxed with stirring for 3 h. The solvent was removed under reduced pressure and solid residue was suspended in EtOH, filtered, and washed with EtOH, EtOAc, and DCM (2 times each) to yield the desired product.

(*Z*)-5-(5-((1-(3-(ethoxycarbonyl)phenyl)-2-oxoindolin-3-ylidene)methyl)furan-2-yl)-2-fluorobenzoic acid (**7a**). Red solid (67% yield). TLC: 5% MeOH in DCM, R_f = 0.1; visualized with UV. ^{1}H NMR (400 MHz, DMSO): δ 8.68 (d, J = 7.7 Hz, 1H), 8.55 (d, J = 6.7 Hz, 1H), 8.26 – 8.18 (m, 1H), 8.06 (d, J = 9.1 Hz, 2H), 7.82 (d, J = 8.0 Hz, 1H), 7.76 (t, J = 7.8 Hz, 1H), 7.55 (d, J = 4.4

Hz, 3H), 7.49 (d, *J* = 3.6 Hz, 1H), 7.36 (t, *J* = 7.7 Hz, 1H), 7.28 (t, *J* = 7.4 Hz, 1H), 6.87 (d, *J* = 7.8 Hz, 1H), 4.36 (q, *J* = 7.1 Hz, 2H), 1.34 (t, *J* = 7.1 Hz, 3H); ^{13}C NMR (101 MHz, DMSO): δ 167.86, 165.47, 164.83, 164.80, 160.38, 156.96, 151.06, 144.12, 141.98, 135.02, 132.15, 131.88, 130.73, 130.42, 130.33, 129.22, 128.27, 128.04, 126.74, 126.01, 123.11, 122.64, 122.22, 121.99, 121.32, 121.20, 119.55, 119.45, 118.53, 118.30, 117.21, 111.85, 110.33, 61.62, 14.60. MS (ESI) *m*/*z* = 496.1 [M - H]$^-$.

(*Z*)-5-(5-((1-(3-(ethoxycarbonyl)phenyl)-2-oxoindolin-3-ylidene)methyl)furan-2-yl)-2-methoxybenzoic acid (**7b**). yellow solid (72% yield). TLC: 5% MeOH in DCM, R_f = 0.2; visualized with UV. ^{1}H NMR (400 MHz, DMSO): δ 8.66 (d, *J* = 5.42 Hz, 1H), 8.34 (s, 1H), 8.09-7.98 (m, 3H), 7.79-7.74 (m, 2H), 7.52 (s, 2H), 7.34.7.25 (m, 4H), 6.86 (d, *J* = 6.97 Hz, 1H), 4.35 (q, *J* = 5.72 Hz, 2H), 3.91 (s, 3H), 1.32 (t, *J* = 9.53 Hz, 3H). ^{13}C NMR (101 MHz, DMSO): δ 177.5, 167.6, 166.7, 165.0, 158.9, 157.1, 150.0, 142.4, 134.9, 131.6, 131.3, 130.2, 129.4, 129.2, 128.4, 127.4, 127.2, 126.7, 125.3, 123.6, 122.5, 122.0, 121.2, 121.1, 120.2, 119.2, 113.7, 113.3, 109.4, 108.9, 107.8, 61.1, 56.1, 14.1. MS (ESI) *m*/*z* = 508.1 [M - H]$^-$.

Synthetic procedure for Amide coupling 9a-9d.

Ethyl (*Z*)-3-(3-((5-(4-methoxy-3-((3-(trifluoromethoxy)phenyl)carbamoyl)phenyl)furan-2-yl)methylene)-2-oxoindolin-1-yl)benzoate (**9a**). To the solution of (*Z*)-5-(5-((1-(3-(ethoxycarbonyl)phenyl)-2-oxoindolin-3-ylidene)methyl)furan-2-yl)-2-methoxybenzoic acid (**7b)** (0.6 g, 1.17 mmol, 1 eq) and HATU (0.672 g, 0.176 mmol, 1.5 eq) in DMF (10 mL) was added DIPEA (0.631 mL, 3.53 mmol, 3 eq), and the mixture was stirred for 10 min at room temperature under an argon atmosphere, then 3-(trifluoromethoxy)aniline (0.173 mL, 1.29 mmol, 1.1 eq) was added. The resultant reaction mixture was stirred at room temperature for 16 h. The reaction mixture was poured into the ice-cold water and extracted with EtOAc (3 × 30 mL). The combined organic extracts were washed with saturated $NaHCO_3$ (2 × 20 mL), followed by brine solution (2 × 20 mL). The combined organic layer was dried over Na_2SO_4 and concentrated under reduced pressure to give crude solid product. The crude product was triturated with 20% EtOAc in hexanes (2-3 times) to afford **9a** (0.61 g, 77% yield) as a red solid. TLC: 50% EtOAc in hexanes, R_f = 0.5; visualized with UV. ^{1}H NMR (400 MHz, DMSO): δ 10.55 (s, 1H), 8.66 (d, *J* = 8.61 Hz, 1H), 8.34-8.26 (m, 1H), 8.12-8.00 (m, 3H), 7.99-7.94 (m, 1H), 7.80-7.78 (m, 1H), 7.75-7.71 (m, 1H),7.67-7.63 (m, 1H), 7.52-7.46 (m, 3H), 7.37-7.32 (m, 2H), 7.29-7.21 (m, 2H), 7.11-7.08 (m, 1H), 6.86-6.83 (m, 1H), 4.35 (q, *J* = 7.02 Hz, 2H), 3.94 (s, 3H), 1.33 (t, *J* = 7.03 Hz, 3H). ^{13}C NMR (101 MHz, DMSO): δ 177.9, 167.5, 165.0, 164.5, 159.0, 157.6, 157.3, 157.2, 157.1, 151.4, 150.1, 148.5, 143.3, 140.6, 134.8, 131.5, 130.5, 130.1, 129.3, 128.8, 128.6, 128.4, 128.0, 127.3, 126.7,

126.2, 125.9, 125.6, 125.3, 123.6, 121.6, 121.2, 121.1, 120.2, 119.3, 118.3, 115.7, 113.6, 112.8, 111.7, 109.6, 108.9, 108.0, 61.1, 56.2, 14.1. LCMS (ESI) *m/z* = 669.1 [M + H]$^+$.

Ethyl (*Z*)-3-(3-((5-(4-fluoro-3-((3-(trifluoromethoxy)phenyl)carbamoyl)phenyl)furan-2-yl)methylene)-2-oxoindolin-1-yl)benzoate (**9b**). Compound **9b** was synthesized by an above synthetic procedure described for the preparation of **9a** using (*Z*)-5-(5-((1-(3-(ethoxycarbonyl)phenyl)-2-oxoindolin-3-ylidene)methyl)furan-2-yl)-2-fluorobenzoic acid (**7a**) (1.8 g, 3.62 mmol, 1 eq) and 3-(trifluoromethoxy)aniline (0.769 g, 4.34 mmol, 1.2 eq) as starting materials. Red solid (1.9 g, 80% yield). TLC: 50% EtOAc in hexanes, R_f = 0.2; visualized with UV. ^{1}H NMR (400 MHz, DMSO): δ 10.95 (s, 1H), 8.65 (d, J = 7.7 Hz, 1H), 8.36 – 8.28 (m, 1H), 8.18 (s, 1H), 8.05 (d, J = 9.4 Hz, 2H), 7.93 (s, 1H), 7.82 (d, J = 7.9 Hz, 1H), 7.75 (dd, J = 13.5, 7.6 Hz, 2H), 7.65 (t, J = 9.2 Hz, 1H), 7.57 (d, J = 2.2 Hz, 2H), 7.54 (d, J = 7.7 Hz, 2H), 7.29 (t, J = 7.6 Hz, 1H), 7.24 – 7.13 (m, 2H), 6.86 (d, J = 7.8 Hz, 1H), 4.36 (q, J = 7.1 Hz, 2H), 1.34 (t, J = 7.2 Hz, 3H). ^{13}C NMR (101 MHz, DMSO). δ 167.96, 165.50, 162.99, 158.28, 156.33, 151.21, 148.98, 143.10, 140.82, 135.27, 132.06, 131.81, 131.20, 130.68, 130.14, 128.97, 127.87, 126.22, 125.98, 125.82, 125.35, 124.23, 123.05, 121.49, 120.72, 120.64, 118.89, 116.66, 112.28, 111.51, 109.54, 61.60, 14.60. LCMS (ESI) *m/z* = 657.1 [M + H]$^+$.

Ethyl (*Z*)-3-(3-((5-(4-methoxy-3-((3-methoxyphenyl)carbamoyl)phenyl)furan-2-yl)methylene)-2-oxoindolin-1-yl)benzoate (**9c**). Compound **9c** was synthesized by an above synthetic procedure described for the preparation of **9a** using (*Z*)-5-(5-((1-(3-(ethoxycarbonyl)phenyl)-2-oxoindolin-3-ylidene)methyl)furan-2-yl)-2-methoxybenzoic acid (**7b**) (0.6 g, 1.17 mmol, 1 eq) and 3-methoxyaniline (0.146 mL, 1.29 mmol, 1.1 eq) as starting materials. Red solid (0.66 g, 91% yield). TLC: 50% EtOAc in hexanes, R_f = 0.4; visualized with UV. ^{1}H NMR (400 MHz, DMSO): δ 10.25 (s, 1H), 8.68 (d, *J* = 8.97 Hz, 1H), 8.09-8.06 (m, 1H), 8.04-8.02 (m, 2H), 7.97-7.95 (m, 1H), 7.80-7.78 (m, 1H), 7.73 (t, *J* = 7.85 Hz, 1H), 7.52-7.51 (m, 1H), 7.49-7.47 (m, 1H), 7.41 (d, *J* = 9.15 Hz, 1H), 7.37 (d, *J* = 3.92 Hz, 1H), 7.31-7.28 (m, 2H), 7.26-7.24 (m, 2H), 6.86-6.85 (m, 1H), 6.72-6.67 (m, 1H), 4.35 (q, *J* = 7.71 Hz, 2H), 3.99 (s, 3H), 3.77 (s, 3H), 1.33 (t, *J* = 7.10 Hz, 3H). ^{13}C NMR (101 MHz, DMSO): δ 177.5, 167.6, 165.0, 164.1, 163.9, 160.0, 159.5, 157.7, 157.2, 156.6, 155.3, 152.7, 151.4, 151.0, 150.0, 148.0, 142.3, 140.1, 134.8, 131.5, 131.3, 130.1, 129.9, 129.5, 129.4, 128.4, 127.9, 127.4, 126.2, 125.9, 125.6, 125.5, 125.3, 123.6, 122.6, 122.0, 121.6, 121.2, 120.2, 119.3, 113.3, 112.8, 112.0, 111.9, 109.6, 108.9, 107.7, 106.3, 105.5, 105.4, 61.1, 56.3, 55.1, 14.1. LCMS (ESI) *m/z* = 615.1 [M + H]$^+$.

Ethyl (*Z*)-3-(3-((5-(4-fluoro-3-((3-methoxyphenyl)carbamoyl)phenyl)furan-2-yl)methylene)-2-oxoindolin-1-yl)benzoate (**9d**). Compound **9d** was synthesized by an above synthetic procedure

described for the preparation of **9a** using (*Z*)-5-(5-((1-(3-(ethoxycarbonyl)phenyl)-2-oxoindolin-3-ylidene)methyl)furan-2-yl)-2-fluorobenzoic acid (**7a**) (0.6 g, 1.20 mmol, 1 eq) and 3-methoxyaniline (0.163 mL, 1.44 mmol, 1.2 eq) as starting materials. Red solid (0.59 g, 81% yield). TLC: 50% EtOAc in hexanes, R_f = 0.4; visualized with UV. Isomer data: ^{1}H NMR (400 MHz, DMSO): δ 10.63 (s, 1H), 8.66 (d, J = 7.7 Hz, 1H), 8.29 (d, J = 6.4 Hz, 1H), 8.16 (t, J = 6.5 Hz, 1H), 8.09 – 8.01 (m, 2H), 7.82 (d, J = 8.0 Hz, 1H), 7.76 (t, J = 7.4 Hz, 1H), 7.63 (t, J = 9.0 Hz, 1H), 7.57 (d, J = 2.2 Hz, 2H), 7.55 – 7.50 (m, 1H), 7.46 (s, 1H), 7.37 – 7.26 (m, 3H), 7.21 (t, J = 7.3 Hz, 1H), 6.86 (d, J = 7.9 Hz, 1H), 6.75 (d, J = 7.8 Hz, 1H), 4.35 (dd, J = 7.9, 5.9 Hz, 2H), 3.77 (d, J = 1.9 Hz, 3H). ^{13}C NMR (101 MHz, DMSO) δ 167.97, 165.51, 162.61, 160.03, 156.43, 151.18, 143.09, 140.38, 135.28, 132.07, 131.82, 130.69, 130.19, 128.97, 127.88, 126.32, 126.15, 125.38, 123.08, 121.49, 120.68, 112.53, 111.46, 109.98, 109.54, 106.03, 61.60, 55.53, 14.61. LCMS (ESI) m/z = 603.1 $[M + H]^+$.

Synthesis of target compounds GL-3392, GL-3618, GL-3395 and GL-3649.

Synthesis of (*Z*)-3-(3-((5-(4-methoxy-3-((3-(trifluoromethoxy)phenyl)carbamoyl)phenyl)furan-2-yl)methylene)-2-oxoindolin-1-yl)benzoic acid (**GL-3392**). To a stirred suspension of ester **9a** (0.3 g, 0.448 mmol, 1 eq) in THF:H_2O (2:1, 3 mL) was added LiOH (0.107 g, 4.48 mmol, 1 eq). The reaction mixture was stirred at room temperature for 16 h. The solvent was removed under reduced pressure to get solid residue. The residue was diluted with water and acidified with 20% citric acid solution to adjust pH 2-3 then extracted with EtOAc (3 × 10 mL). The combined organic extracts were washed with brine, dried over Na_2SO_4, and concentrated under reduced pressure. The crude product was purified using 2–5% MeOH in DCM (1% AcOH in DCM) solvent system on flash column chromatography. The product was then crystallized in EtOH, and solid was collected, washed with EtOAc and then hot solutions of 20-30% EtOAc in hexanes to give target compound **GL-3392** (0.216 g, 75% yield) as a yellow solid. TLC: 10% MeOH in DCM, R_f = 0.3; visualized with UV. ^{1}H NMR (400 MHz, DMSO): δ 13.3 (bs, 1H), 10.60 (s, 1H), 8.67 (d, J = 6.51 Hz, 1H), 8.25 (d, J = 3.6 Hz, 1H), 8.11 (dd, J = 6.31 Hz, 2.81 Hz, 1H), 8.03-8.02 (m, 1H), 8.01-7.99 (m, 2H), 7.97 (brs, 1H), 7.77-7.75 (m, 1H), 7.73-7.69 (m, 1H), 7.54-7.53 (m, 2H), 7.50 (d, J =7.83 Hz, 1H), 7.44 (d, J = 8.70 Hz, 1H), 7.40 (d, J = 4.06 Hz, 1H), 7.31-7.27 (m, 1H), 7.25-7.23 (m, 1H), 7.13-7.11 (m, 1H), 6.86 (d, J = 6.55 Hz, 1H). 3.98 (s, 3H). ^{13}C NMR (101 MHz, DMSO): δ 167.5, 166.6, 164.5, 157.2, 150.1, 148.5, 142.4, 140.6, 134.7, 131.1, 130.6, 130.0, 129.4, 128.6, 128.0, 127.4, 125.8, 125.6, 125.3, 123.6, 123.5, 122.5, 121.6, 121.2, 119.3, 118.3, 115.7, 113.2, 111.7, 109.6, 109.0, 56.3. ^{19}F NMR (376 MHz, DMSO): δ -56.63 (s, 3F). LCMS (ESI) m/z = 639.15 $[M - H]^-$. HPLC purity: 99.21% (t_R = *Z* isomer at 11.29 min and *E* isomer at 11.67 min).

<u>(*Z*)-3-(3-((5-(4-fluoro-3-((3-(trifluoromethoxy)phenyl)carbamoyl)phenyl)furan-2-yl)methylene)-2-oxoindolin-1-yl)benzoic acid (**GL-3618**).</u> Target compound **GL-3618** was synthesized by an above synthetic procedure described for the preparation of **GL-3392** using ethyl (*Z*)-3-(3-((5-(4-fluoro-3-((3-(trifluoromethoxy)phenyl)carbamoyl)phenyl)furan-2-yl)methylene)-2-oxoindolin-1-yl)benzoate **9b** (0.02 g, 0.03 mmol, 1 eq) as starting materials. Yellow solid (0.015 g, 78% yield). TLC: 10% MeOH in DCM, R_f = 0.3; visualized with UV. ^{1}H NMR (400 MHz, DMSO): δ 13.24 (brs, 1H), 10.92 (s, 1H), 8.65 (d, *J* = 9.03 Hz, 1H), 8.31 (d, *J* = 5.75 Hz, 1H), 8.17 (brs, 1H), 8.03 – 8.00 (m, 2H), 7.91 (s, 1H), 7.86 – 7.72 (m, 3H), 7.63 (t, *J* = 9.85 Hz, 1H), 7.56 – 7.51 (m, 4H), 7.28 (t, *J* = 7.39 Hz, 1H), 7.20 – 7.14 (m, 2H), 6.86 (d, *J* = 8.21 Hz, 1H). ^{13}C NMR (101 MHz, DMSO): δ 167.4, 166.6, 162.5, 155.8, 150.7, 148.5, 142.6, 140.3, 134.6, 131.1, 130.7, 130.0, 129.6, 128.6, 128.5, 127.4, 125.7, 125.5, 124.8, 123.7, 122.5, 121.0, 120.3, 120.1, 118.4, 117.6, 116.1, 111.8, 111.0, 109.0, 61.1. ^{19}F NMR (376 MHz, DMSO): δ -56.67 (s, 3F), -113.1 (s, 1F). LCMS (ESI) *m*/*z* = 627.15 [M - H]$^-$. HPLC purity: 96.52% (t_R = *Z* isomer at 10.77 min and *E* isomer at 11.25 min).

<u>(*Z*)-3-(3-((5-(4-methoxy-3-((3-methoxyphenyl)carbamoyl)phenyl)furan-2-yl)methylene)-2-oxoindolin-1-yl)benzoic acid (**GL-3395**).</u> Target compound **GL-3395** was synthesized by an above synthetic procedure described for the preparation of **GL-3392** using ethyl (*Z*)-3-(3-((5-(4-methoxy-3-((3-methoxyphenyl)carbamoyl)phenyl)furan-2-yl)methylene)-2-oxoindolin-1-yl)benzoate **9c** (0.2 g, 0.32 mmol, 1 eq) as starting materials. Yellow solid (0.292 g, 87% yield). TLC: 10% MeOH in DCM, R_f = 0.2; visualized with UV. ^{1}H NMR (400 MHz, DMSO): δ 10.29 (s, 1H), 8.26 (d, *J* = 3.77 Hz, 1H), 8.09 (dd, *J* = 8.28 Hz, 2.26 Hz, 1H), 8.02-7.98 (m, 2H), 7.72-7.68 (m, 2H), 7.54 (s, 2H), 7.48 (s, 1H), 7.43-7.39 (m, 2H), 7.34-7.23 (m, 4H), 6.85 (d, *J* = 7.53 Hz, 1H), 6.70 (dd, *J* = 8.23 Hz, 1.75 Hz, 1H), 3.98 (s, 3H), 3.76 (s, 3H). ^{13}C NMR (101 MHz, DMSO): δ 167.5, 166.9, 163.9, 159.5, 157.2, 150.1, 142.5, 140.1, 134.6, 133.6, 130.6, 129.7, 129.6, 129.4, 128.5, 127.9, 127.4, 125.9, 125.6, 125.2, 123.6, 122.5, 121.6, 120.1, 119.3, 113.2, 112.2, 109.5, 109.1, 109.0, 105.5, 56.3, 55.0. LCMS (ESI) *m*/*z* = 585.15 [M - H]$^-$. HPLC purity: 98.18% (t_R = *Z* isomer at 10.33 min and *E* isomer at 10.76 min).

<u>(*Z*)-3-(3-((5-(4-fluoro-3-((3-methoxyphenyl)carbamoyl)phenyl)furan-2-yl)methylene)-2-oxoindolin-1-yl)benzoic acid (**GL-3649**).</u> Target compound **GL-3649** was synthesized by an above synthetic procedure described for the preparation of **GL-3392** using ethyl (*Z*)-3-(3-((5-(4-fluoro-3-((3-methoxyphenyl)carbamoyl)phenyl)furan-2-yl)methylene)-2-oxoindolin-1-yl)benzoate **9d** (0.3 g, 0.49 mmol, 1 eq) as starting materials. Yellow solid (0.220 g, 76% yield). TLC: 10% MeOH in DCM, R_f = 0.2; visualized with UV. ^{1}H NMR (400 MHz, DMSO): δ 13.2 (brs, 1H), 10.63 (s, 1H), 8.64 (d, *J* = 7.37 Hz, 1H), 8.27 (brs, 1H), 8.14 (brs, 1H), 8.03 – 8.00 (m, 2H), 7.75 – 7.71 (m, 2H),

7.61 - 7.47 (m, 5H), 7.32 – 7.29 (m, 3H), 7.21 – 7.19 (m, 1H), 6.85 (d, J = 7.37 Hz, 1H), 6.73 (d, J = 7.37 Hz, 1H), 3.76 (s, 3H). ^{13}C NMR (101 MHz, DMSO): δ 167.5, 166.6, 162.1, 159.9, 159.5, 157.8, 155.9, 150.7, 142.6, 139.9, 139.3, 133.5, 134.6, 132.4, 131.1, 130.0, 129.7, 128.6, 128.2, 127.4, 126.0, 125.7, 124.9, 123.7, 122.6, 121.0, 120.2, 117.8, 112.0, 111.0, 109.5, 109.1, 105.5, 55.0. ^{19}F NMR (376 MHz, DMSO): δ -113.1 (s, 1F). LCMS (ESI) m/z = 573.15 [M - H]$^{-}$.

2) Proton (1H), Carbon (13C), APT and Fluorine (19F) NMR spectra of Target compounds:

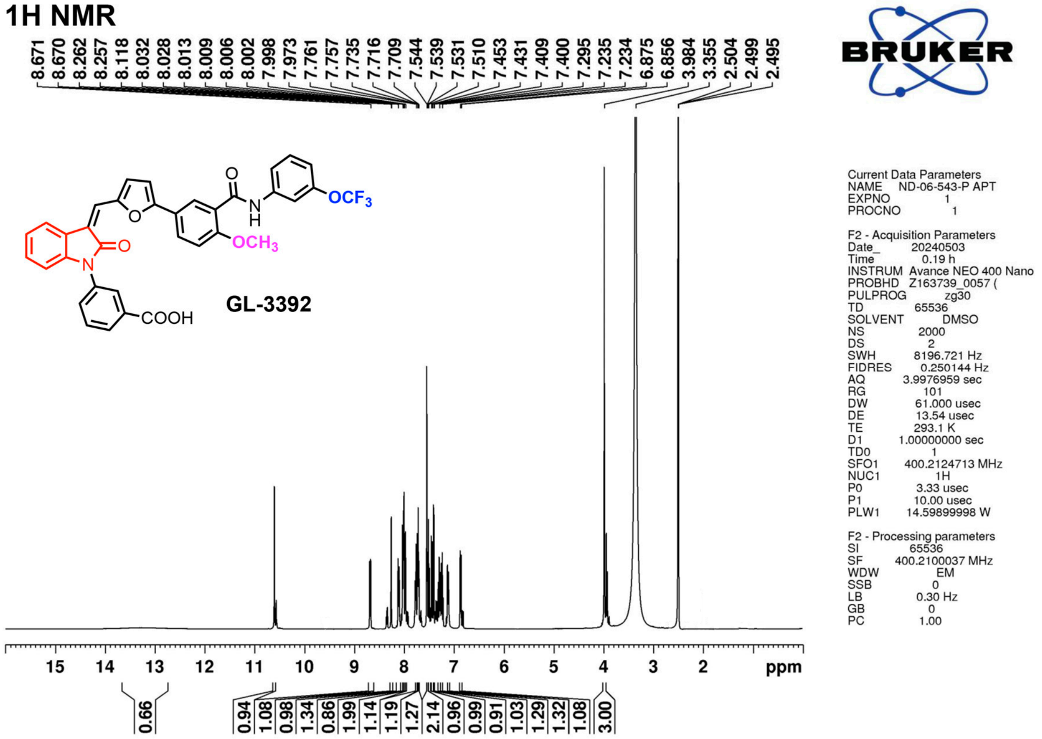

13C NMR

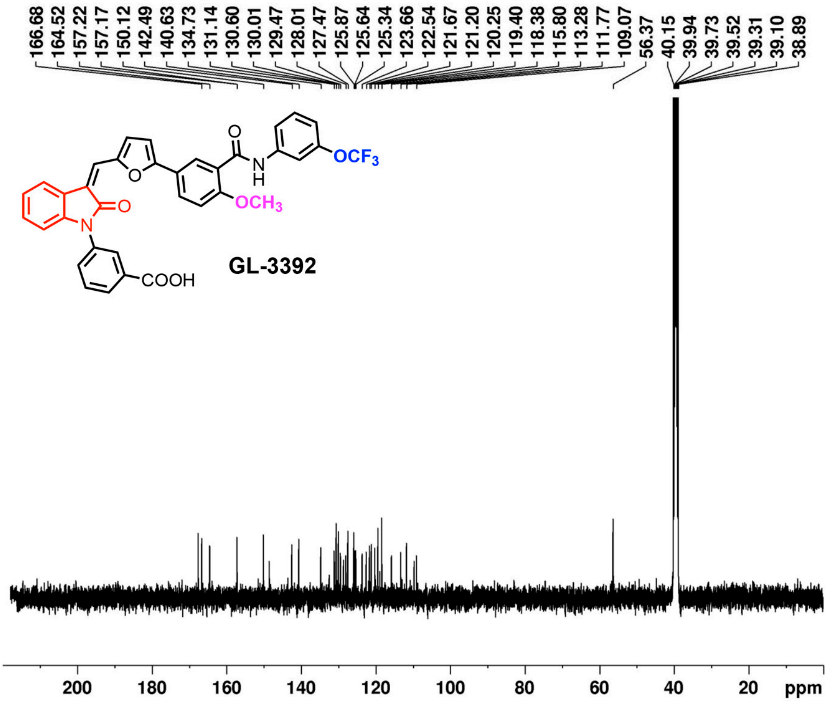

Current Data Parameters
NAME ND-06-543 in DMSO
EXPNO 2
PROCNO 1

F2 - Acquisition Parameters
Date_ 20240425
Time 23.52 h
INSTRUM Avance NEO 400 Nano
PROBHD Z163739_0057 (
PULPROG zgpg30
TD 65536
SOLVENT DMSO
NS 1500
DS 4
SWH 23809.524 Hz
FIDRES 0.726609 Hz
AQ 1.3762560 sec
RG 101
DW 21.000 usec
DE 6.50 usec
TE 293.5 K
D1 2.00000000 sec
D11 0.03000000 sec
TD0 1
SFO1 100.6429478 MHz
NUC1 13C
P0 3.33 usec
P1 10.00 usec
PLW1 54.87099838 W
SFO2 400.2116008 MHz
NUC2 1H
CPDPRG[2 waltz65
PCPD2 90.00 usec
PLW2 14.59899999 W
PLW12 0.18024001 W
PLW13 0.09065800 W

F2 - Processing parameters
SI 32768
SF 100.6329288 MHz
WDW EM
SSB 0
LB 1.00 Hz
GB 0
PC 1.40

APT NMR

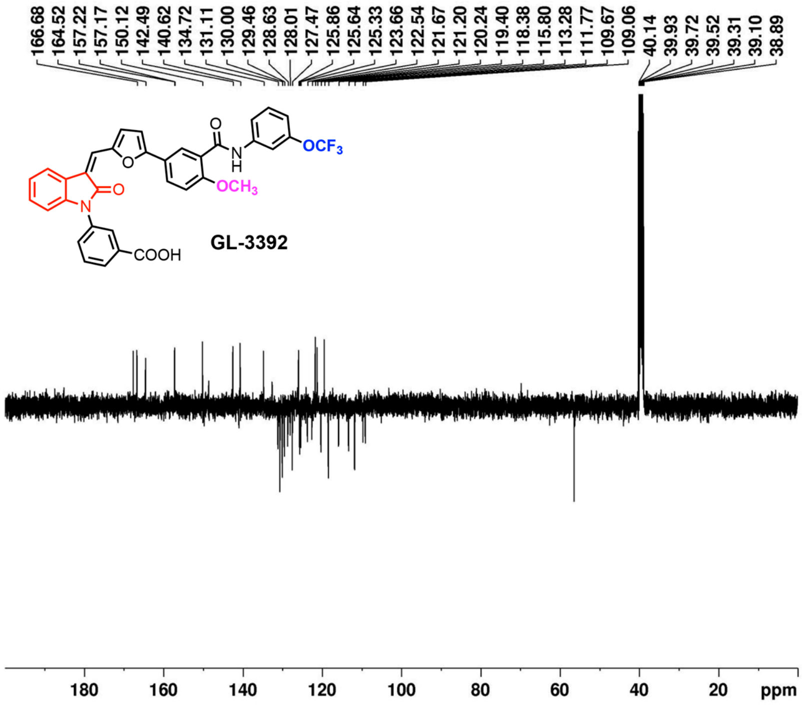

Current Data Parameters
NAME ND-06-543-P APT
EXPNO 2
PROCNO 1

F2 - Acquisition Parameters
Date_ 20240503
Time 1.17 h
INSTRUM Avance NEO 400 Nano
PROBHD Z163739_0057 (
PULPROG jmod
TD 65536
SOLVENT DMSO
NS 1000
DS 4
SWH 23809.524 Hz
FIDRES 0.726609 Hz
AQ 1.3762560 sec
RG 101
DW 21.000 usec
DE 6.50 usec
TE 294.1 K
CNST2 145.0000000
CNST11 1.0000000
D1 2.00000000 sec
D20 0.00689655 sec
TD0 1
SFO1 100.6429478 MHz
NUC1 13C
P1 10.00 usec
P2 20.00 usec
PLW1 54.87099838 W
SFO2 400.2116008 MHz
NUC2 1H
CPDPRG[2 waltz65
PCPD2 90.00 usec
PLW2 14.59899998 W
PLW12 0.18024001 W

F2 - Processing parameters
SI 32768
SF 100.6329291 MHz
WDW EM
SSB 0
LB 1.00 Hz
GB 0
PC 1.40

19F NMR

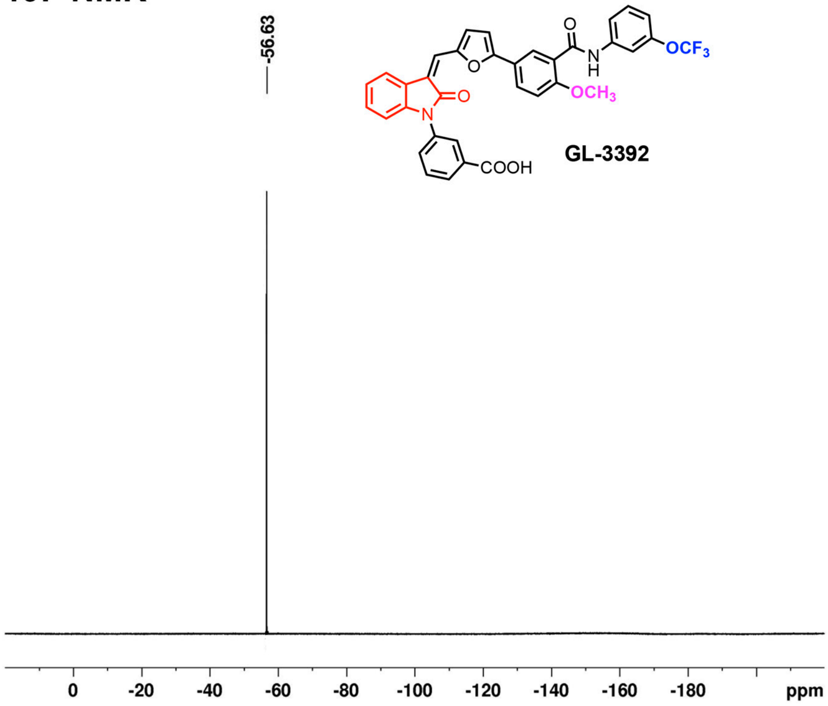

Current Data Parameters
NAME ND-06-543-P in DMSO
EXPNO 2
PROCNO 1

F2 - Acquisition Parameters
Date_ 20240502
Time 12.29 h
INSTRUM Avance NEO 400 Nano
PROBHD Z163739_0057 (
PULPROG zgig
TD 131072
SOLVENT DMSO
NS 16
DS 4
SWH 90909.091 Hz
FIDRES 1.387163 Hz
AQ 0.7208960 sec
RG 101
DW 5.500 usec
DE 6.50 usec
TE 293.8 K
D1 1.00000000 sec
D11 0.03000000 sec
TD0 1
SFO1 376.5359841 MHz
NUC1 19F
P1 18.00 usec
PLW1 13.96000004 W
SFO2 400.2116008 MHz
NUC2 1H
CPDPRG[2 waltz16
PCPD2 90.00 usec
PLW2 14.59899998 W
PLW12 0.18024001 W

F2 - Processing parameters
SI 65536
SF 376.5736414 MHz
WDW EM
SSB 0
LB 0.30 Hz
GB 0
PC 1.00

1H NMR

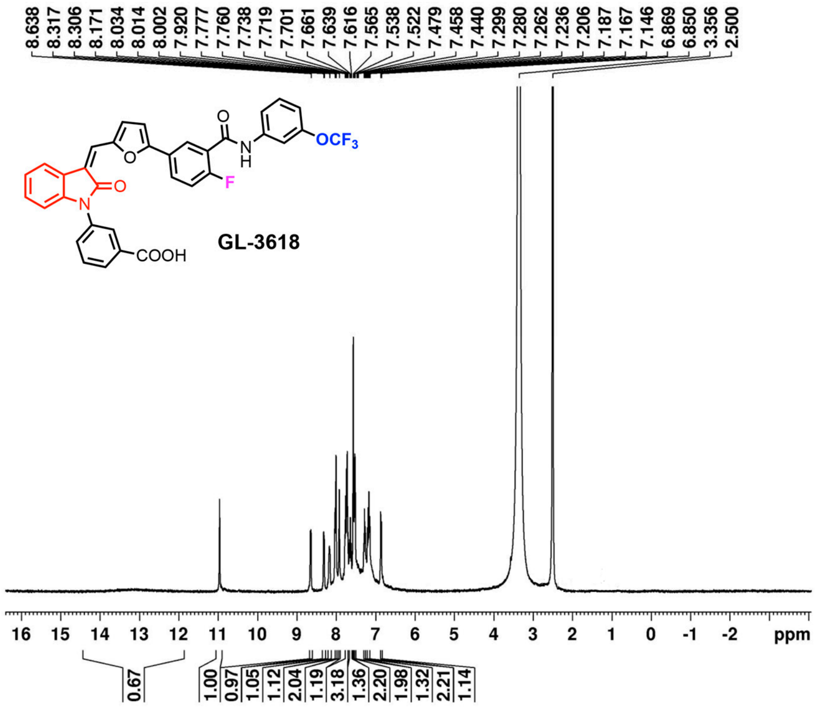

Current Data Parameters
NAME ND-06-544-P in DMSO
EXPNO 1
PROCNO 1

F2 - Acquisition Parameters
Date_ 20240501
Time 10.58 h
INSTRUM Avance NEO 400 Nano
PROBHD Z163739_0057 (
PULPROG zg30
TD 65536
SOLVENT DMSO
NS 16
DS 2
SWH 8196.721 Hz
FIDRES 0.250144 Hz
AQ 3.9976959 sec
RG 101
DW 61.000 usec
DE 13.54 usec
TE 293.8 K
D1 1.00000000 sec
TD0 1
SFO1 400.2124713 MHz
NUC1 1H
P0 3.33 usec
P1 10.00 usec
PLW1 14.59899998 W

F2 - Processing parameters
SI 65536
SF 400.2100037 MHz
WDW EM
SSB 0
LB 0.30 Hz
GB 0
PC 1.00

13C NMR

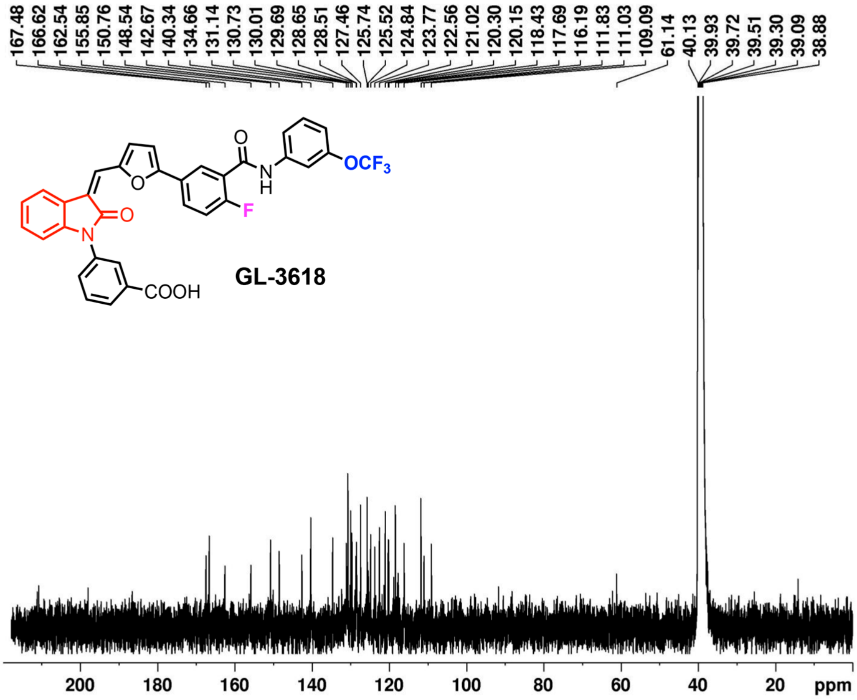

Current Data Parameters
NAME ND-04-389 in DMSO
EXPNO 30
PROCNO 1

F2 - Acquisition Parameters
Date_ 20230929
Time 2.33 h
INSTRUM Avance NEO 400 Nano
PROBHD Z163739_0057 (
PULPROG zgpg30
TD 65536
SOLVENT DMSO
NS 4000
DS 4
SWH 23809.524 Hz
FIDRES 0.726609 Hz
AQ 1.3762560 sec
RG 101
DW 21.000 usec
DE 6.50 usec
TE 298.0 K
D1 2.00000000 sec
D11 0.03000000 sec
TD0 1
SFO1 100.6328888 MHz
NUC1 13C
P0 3.33 usec
P1 10.00 usec
PLW1 56.77000046 W
SFO2 400.1716007 MHz
NUC2 1H
CPDPRG[2 waltz65
PCPD2 90.00 usec
PLW2 15.75399971 W
PLW12 0.19449000 W
PLW13 0.09782900 W

F2 - Processing parameters
SI 32768
SF 100.6228729 MHz
WDW EM
SSB 0
LB 1.00 Hz
GB 0
PC 1.40

APT NMR

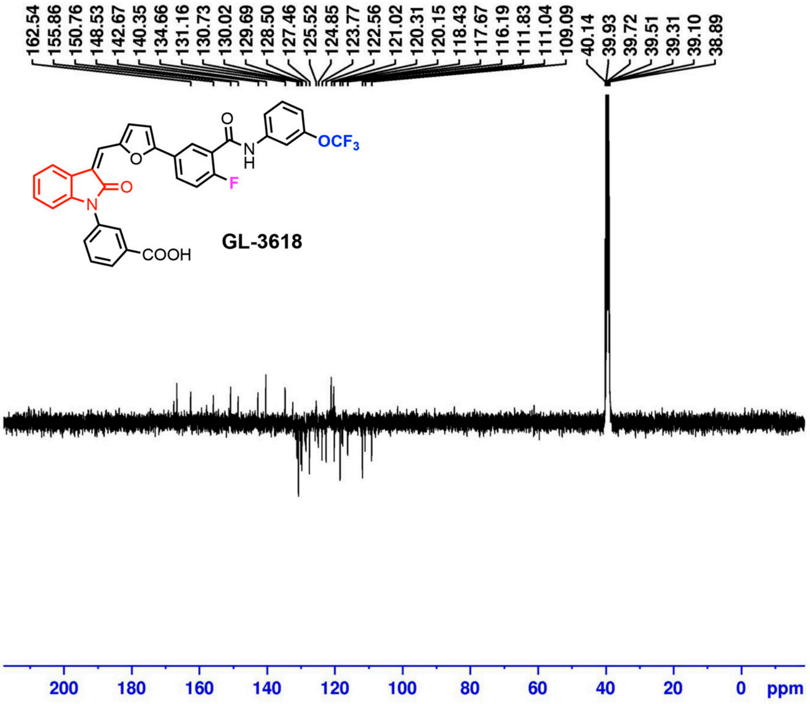

Current Data Parameters
NAME ND-04-389-P in DMSO
EXPNO 21
PROCNO 1

F2 - Acquisition Parameters
Date_ 20230922
Time 0.25 h
INSTRUM Avance NEO 400 Nano
PROBHD Z163739_0057 (
PULPROG jmod
TD 65536
SOLVENT DMSO
NS 1256
DS 4
SWH 23809.524 Hz
FIDRES 0.726609 Hz
AQ 1.3762560 sec
RG 101
DW 21.000 usec
DE 6.50 usec
TE 298.0 K
CNST2 145.0000000
CNST11 1.0000000
D1 2.00000000 sec
D20 0.00689655 sec
TD0 1
SFO1 100.6328888 MHz
NUC1 13C
P1 10.00 usec
P2 20.00 usec
PLW1 56.77000046 W
SFO2 400.1716007 MHz
NUC2 1H
CPDPRG[2 waltz65
PCPD2 90.00 usec
PLW2 15.75399971 W
PLW12 0.19449000 W

F2 - Processing parameters
SI 32768
SF 100.6228729 MHz
WDW EM
SSB 0
LB 1.00 Hz
GB 0
PC 1.40

19F NMR

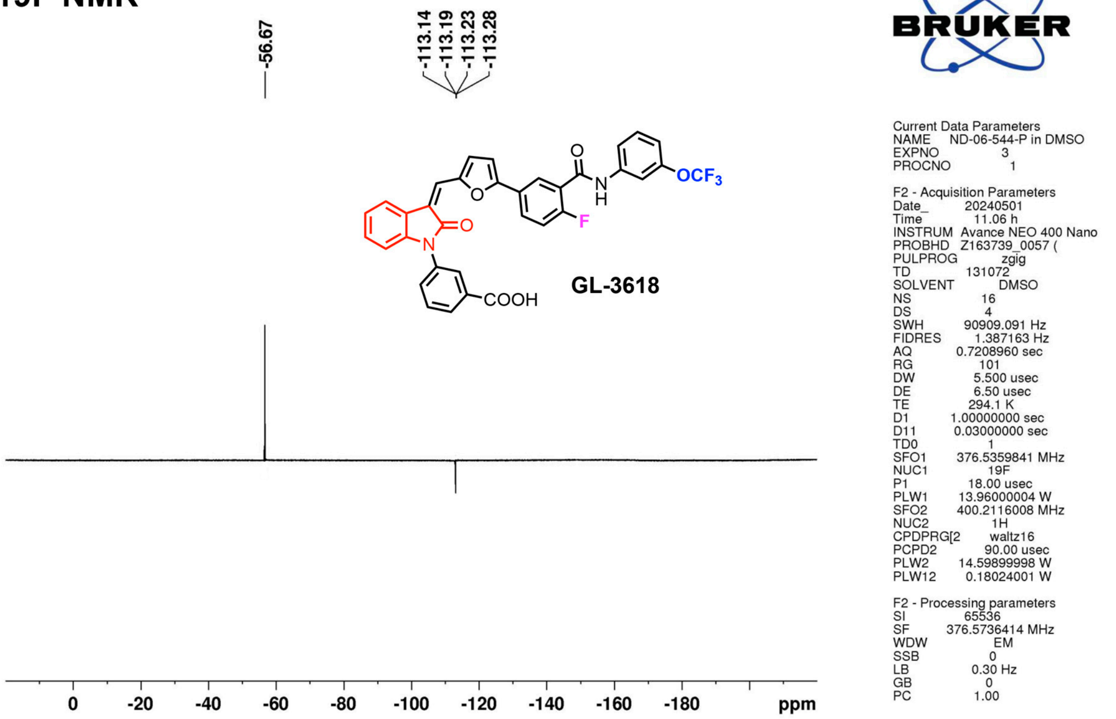

1H NMR

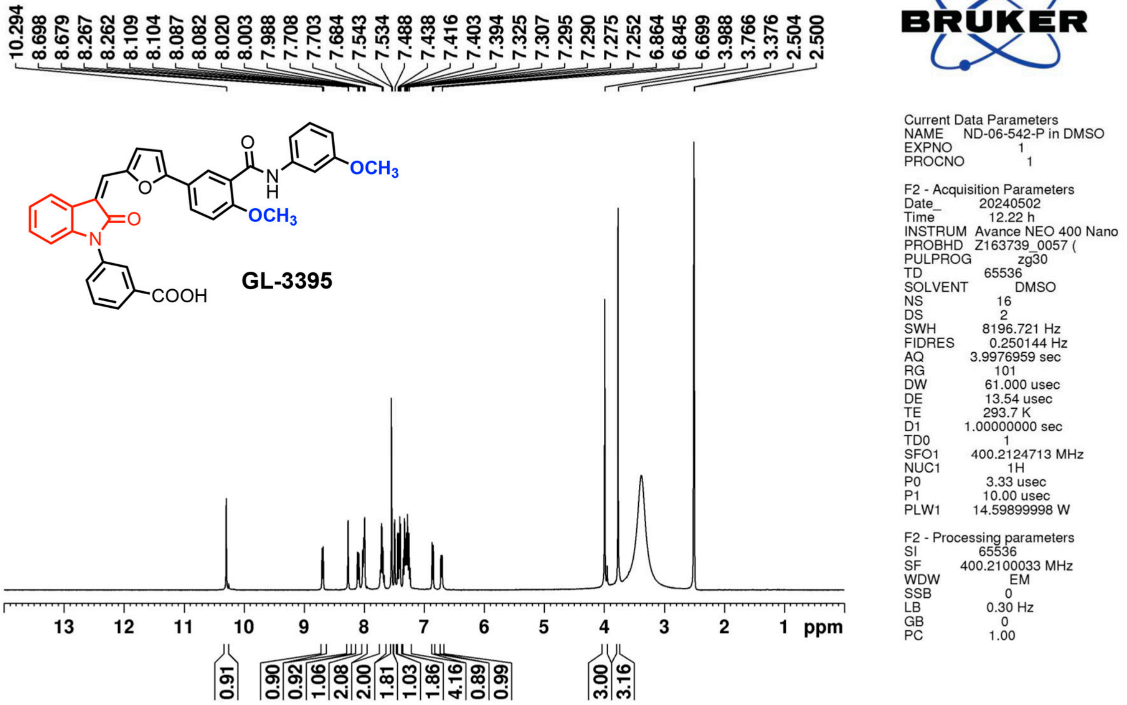

13C NMR

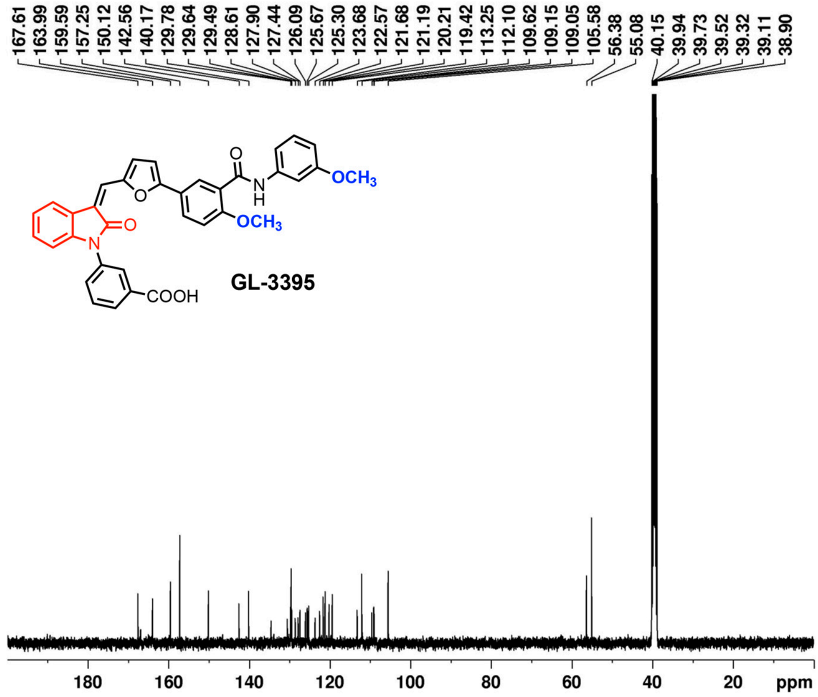

BRUKER

Current Data Parameters
NAME ND-06-542-P APT
EXPNO 1
PROCNO 1

F2 - Acquisition Parameters
Date_ 20240502
Time 20.28 h
INSTRUM Avance NEO 400 Nano
PROBHD Z163739_0057 (
PULPROG zgpg30
TD 65536
SOLVENT DMSO
NS 1024
DS 4
SWH 23809.524 Hz
FIDRES 0.726609 Hz
AQ 1.3762560 sec
RG 101
DW 21.000 usec
DE 6.50 usec
TE 294.0 K
D1 2.00000000 sec
D11 0.03000000 sec
TD0 1
SFO1 100.6429478 MHz
NUC1 13C
P0 3.33 usec
P1 10.00 usec
PLW1 54.87099838 W
SFO2 400.2116008 MHz
NUC2 1H
CPDPRG[2 waltz65
PCPD2 90.00 usec
PLW2 14.59899998 W
PLW12 0.18024001 W
PLW13 0.09065800 W

F2 - Processing parameters
SI 32768
SF 100.6329272 MHz
WDW EM
SSB 0
LB 1.00 Hz
GB 0
PC 1.40

APT NMR

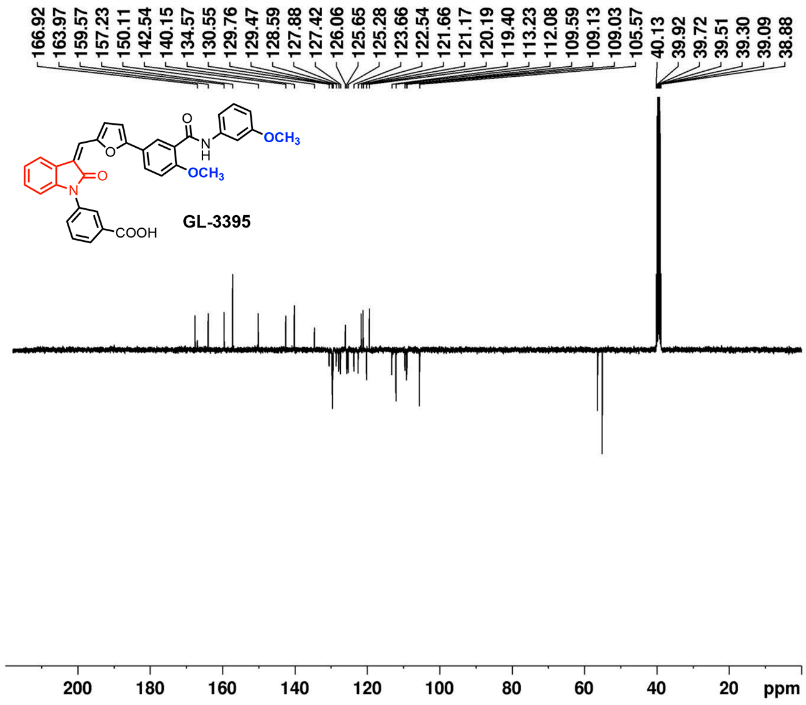

BRUKER

Current Data Parameters
NAME ND-06-542-P APT
EXPNO 2
PROCNO 1

F2 - Acquisition Parameters
Date_ 20240502
Time 21.27 h
INSTRUM Avance NEO 400 Nano
PROBHD Z163739_0057 (
PULPROG jmod
TD 65536
SOLVENT DMSO
NS 1024
DS 4
SWH 23809.524 Hz
FIDRES 0.726609 Hz
AQ 1.3762560 sec
RG 101
DW 21.000 usec
DE 6.50 usec
TE 294.3 K
CNST2 145.0000000
CNST11 1.0000000
D1 2.00000000 sec
D20 0.00689655 sec
TD0 1
SFO1 100.6429478 MHz
NUC1 13C
P1 10.00 usec
P2 20.00 usec
PLW1 54.87099838 W
SFO2 400.2116008 MHz
NUC2 1H
CPDPRG[2 waltz65
PCPD2 90.00 usec
PLW2 14.59899998 W
PLW12 0.18024001 W

F2 - Processing parameters
SI 32768
SF 100.6329293 MHz
WDW EM
SSB 0
LB 1.00 Hz
GB 0
PC 1.40

1H NMR

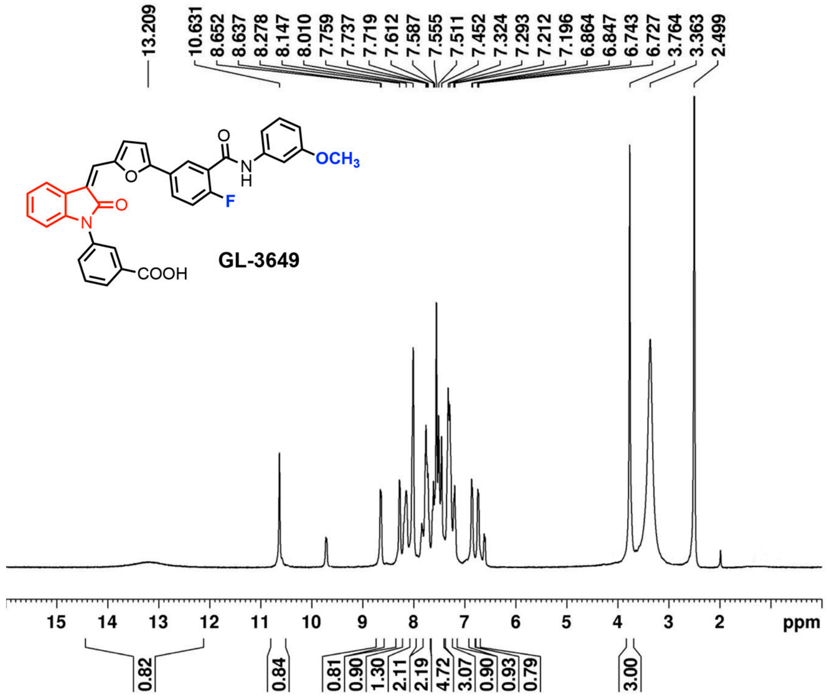

Current Data Parameters
NAME ND-06-517-C13 NMR
EXPNO 1
PROCNO 1

F2 - Acquisition Parameters
Date_ 20240401
Time 19.13 h
INSTRUM Avance NEO 400 Nano
PROBHD Z163739_0057 (
PULPROG zg30
TD 65536
SOLVENT DMSO
NS 16
DS 2
SWH 8196.721 Hz
FIDRES 0.250144 Hz
AQ 3.9976959 sec
RG 101
DW 61.000 usec
DE 13.54 usec
TE 292.6 K
D1 1.00000000 sec
TD0 1
SFO1 400.2124713 MHz
NUC1 1H
P0 3.33 usec
P1 10.00 usec
PLW1 14.59899998 W

F2 - Processing parameters
SI 65536
SF 400.2100037 MHz
WDW EM
SSB 0
LB 0.30 Hz
GB 0
PC 1.00

13C NMR

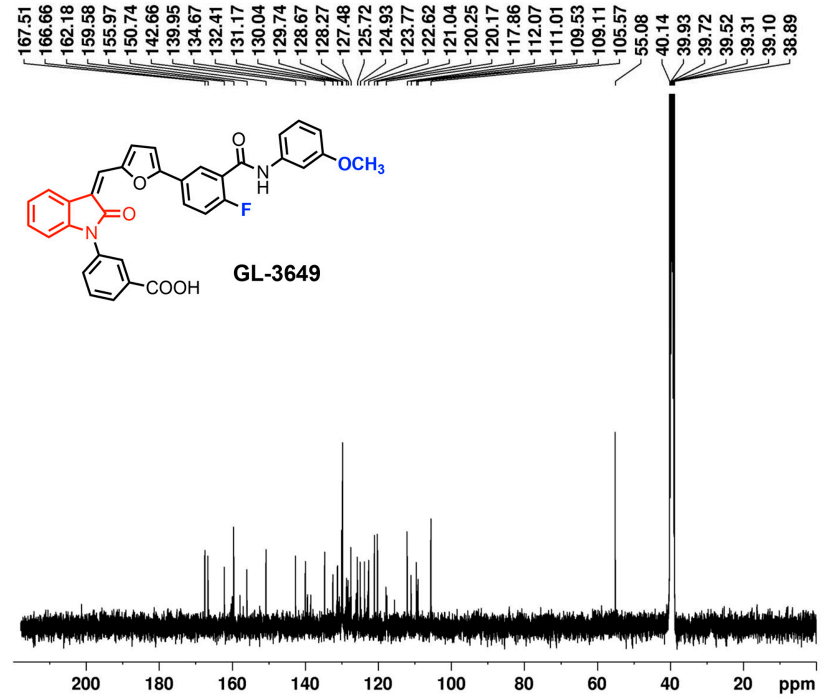

Current Data Parameters
NAME ND-06-517-C13 NMR
EXPNO 4
PROCNO 1

F2 - Acquisition Parameters
Date_ 20240401
Time 20.16 h
INSTRUM Avance NEO 400 Nano
PROBHD Z163739_0057 (
PULPROG zgpg30
TD 65536
SOLVENT DMSO
NS 1024
DS 4
SWH 23809.524 Hz
FIDRES 0.726609 Hz
AQ 1.3762560 sec
RG 101
DW 21.000 usec
DE 6.50 usec
TE 293.5 K
D1 2.00000000 sec
D11 0.03000000 sec
TD0 1
SFO1 100.6429478 MHz
NUC1 13C
P0 3.33 usec
P1 10.00 usec
PLW1 54.87099838 W
SFO2 400.2116008 MHz
NUC2 1H
CPDPRG[2 waltz65
PCPD2 90.00 usec
PLW2 14.59899998 W
PLW12 0.18024001 W
PLW13 0.09065800 W

F2 - Processing parameters
SI 32768
SF 100.6329284 MHz
WDW EM
SSB 0
LB 1.00 Hz
GB 0
PC 1.40

APT NMR

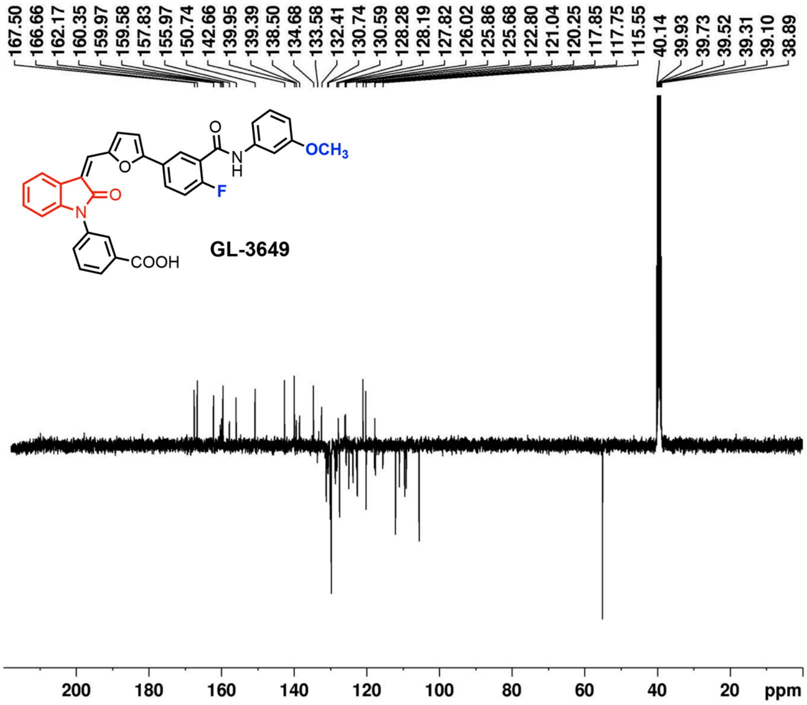

Current Data Parameters
NAME ND-06-517-C13 NMR
EXPNO 5
PROCNO 1

F2 - Acquisition Parameters
Date_ 20240401
Time 21.16 h
INSTRUM Avance NEO 400 Nano
PROBHD Z163739_0057 (
PULPROG jmod
TD 65536
SOLVENT DMSO
NS 1024
DS 4
SWH 23809.524 Hz
FIDRES 0.726609 Hz
AQ 1.3762560 sec
RG 101
DW 21.000 usec
DE 6.50 usec
TE 293.9 K
CNST2 145.0000000
CNST11 1.0000000
D1 2.00000000 sec
D20 0.00689655 sec
TD0 1
SFO1 100.6429478 MHz
NUC1 13C
P1 10.00 usec
P2 20.00 usec
PLW1 54.87099838 W
SFO2 400.2116008 MHz
NUC2 1H
CPDPRG[2 waltz65
PCPD2 90.00 usec
PLW2 14.59899998 W
PLW12 0.18024001 W

F2 - Processing parameters
SI 32768
SF 100.6329286 MHz
WDW EM
SSB 0
LB 1.00 Hz
GB 0
PC 1.40

19F NMR

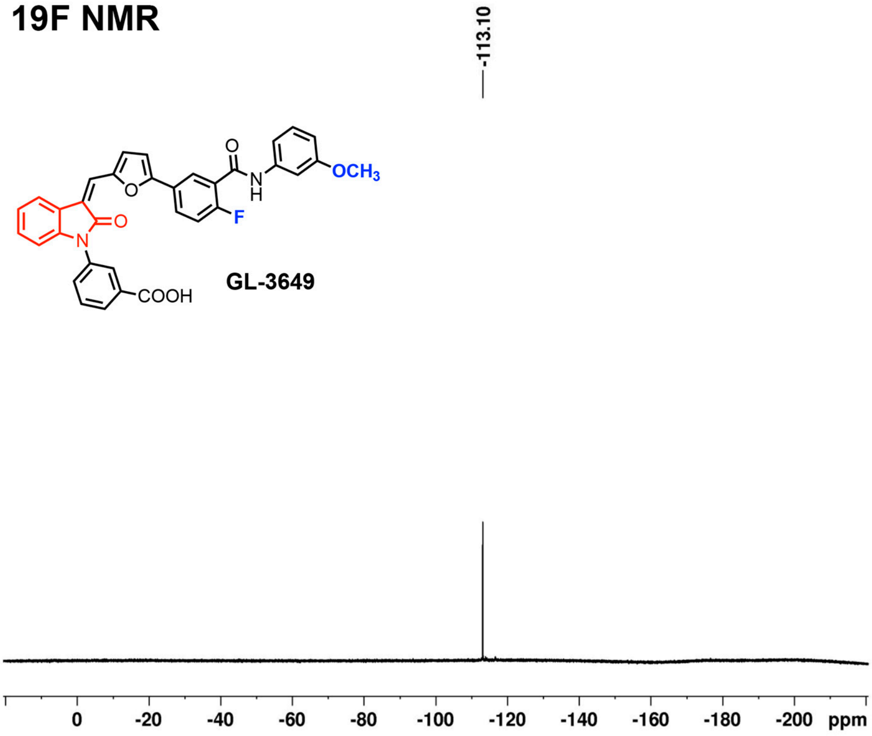

Current Data Parameters
NAME ND-06-517-C13 NMR
EXPNO 2
PROCNO 1

F2 - Acquisition Parameters
Date_ 20240401
Time 19.14 h
INSTRUM Avance NEO 400 Nano
PROBHD Z163739_0057 (
PULPROG zg
TD 131072
SOLVENT DMSO
NS 16
DS 4
SWH 90909.091 Hz
FIDRES 1.387163 Hz
AQ 0.7208960 sec
RG 101
DW 5.500 usec
DE 6.50 usec
TE 292.6 K
D1 1.00000000 sec
TD0 1
SFO1 376.5359841 MHz
NUC1 19F
P1 18.00 usec
PLW1 13.96000004 W

F2 - Processing parameters
SI 65536
SF 376.5736414 MHz
WDW EM
SSB 0
LB 0.30 Hz
GB 0
PC 1.00

Supplementary Table S1. Chemical Properties of Novel Ku-DBis. TPSA is predicted/calculated topological polar surface area and CLogP is predicted/calculated octanol/water partition co-efficient (LogP) using QuikProp (v5.1, Schrödinger Suite 2024). TPSA <140 Å2 appear to achieve good cell permeability and oral bioavailability. Calculated CLogP values are commonly used in analyses describing the influence of lipophilicity on drug properties (CLogP < 3 means less lipophilic and CLogP > 3, more lipophilic compound). The greater the lipophilicity of a drug, tends to lead to higher cell permeability.

Chemical Properties	GL-3392	GL-3618	GL-3395	GL-3649
TPSA	114.4	105.17	114.4	105.17
CLogP	6.52	5.98	6.17	5.30

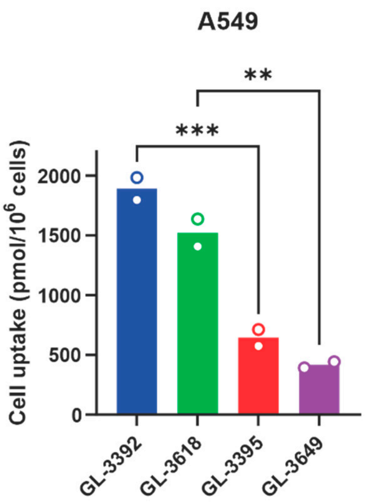

Supplementary Figure S2. Cellular uptake of Ku-DBis assessed in A549 NSCLC cells. A549 cells were incubated with 10 μM Ku-DBi for 4h. After incubation, extracted samples in methanol were analyzed by HPLC, and picomoles of compound per million cells were calculated. Data are presented as the mean of duplicate determinations. ***p =0.0009, **p=0.0014 as calculated by one-way ANOVA with Šídák's multiple comparisons tests.

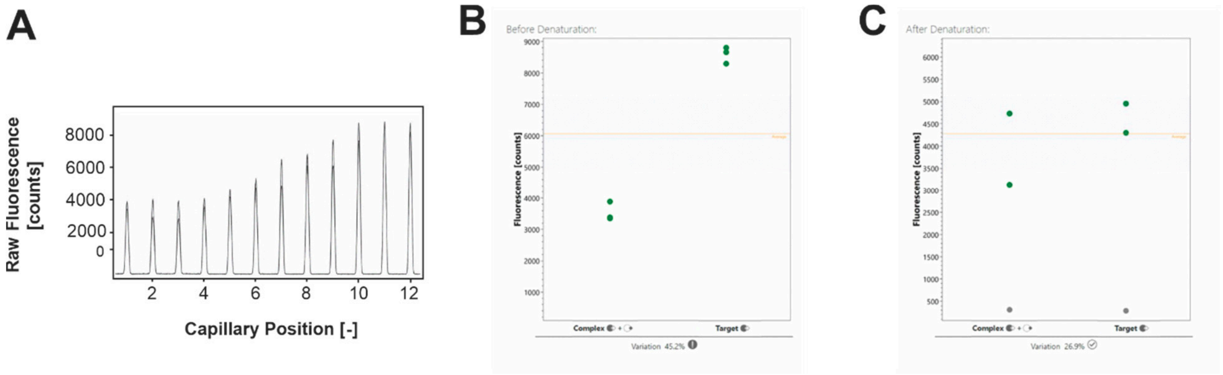

Supplementary Figure S3. Raw Initial Fluorescence and SDS Denaturation Test (SD-Test). (A) Measurement of raw initial fluorescence of protein in the presence of ligand **3392**: concentration range from 98nM to 200 □M in a 1:1 dilution series of 12 points. (B) Raw initial fluorescence before denaturation of complex form (capillaries n°1, n°2, n°3) and Apo form (capillaries n°10, n°11, n°12).(C) Raw initial fluorescence after denaturation. Denaturation consisted of centrifuging the remaining tubes (1 to 3 and 10 to 12) prepared in the original binding assay for at least 10 minutes at =15,000g, carefully removing the supernatants, mixing each with SD-mix (4% SDS, 40 mM DTT), and finally incubating for 5 minutes at 95°C to denature the protein.

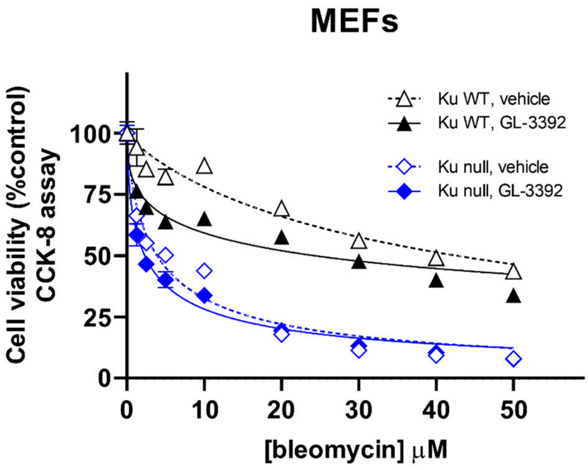

Supplementary Figure S4. Ku-DBi 3392 sensitivity to bleomycin in wild-type (WT) and Ku 80-null MEFs cells. MEF cells were pre-treated with vehicle or 20 μM **3392** for 24 h and then increasing concentrations of bleomycin for 48 h after which cell viability was determined by CCK-8. Data are presented as the mean and SEM of triplicate determinations.

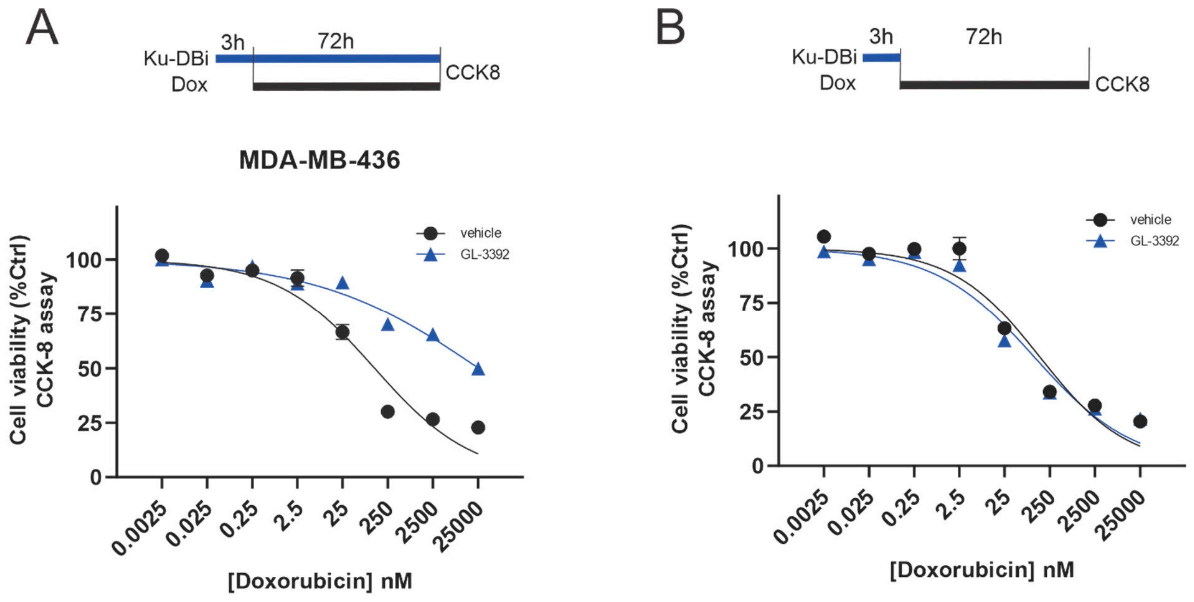

Supplementary **Figure S5. Decreased sensitivity to Doxorubicin mediated by Ku-DBi in MDA-436 cells.** (A) Cells were pre-treated for 3 h with vehicle or 20 □M **3392** prior to 72 h treatment with increasing concentrations of Doxorubicin. (B) Pre-treatment with vehicle or 20 □M **3392** and media removed after 3 h, prior 72 h doxorubicin treatment after which cell viability was determined by CCK-8. Data are presented as the mean and SEM of triplicate determinations.

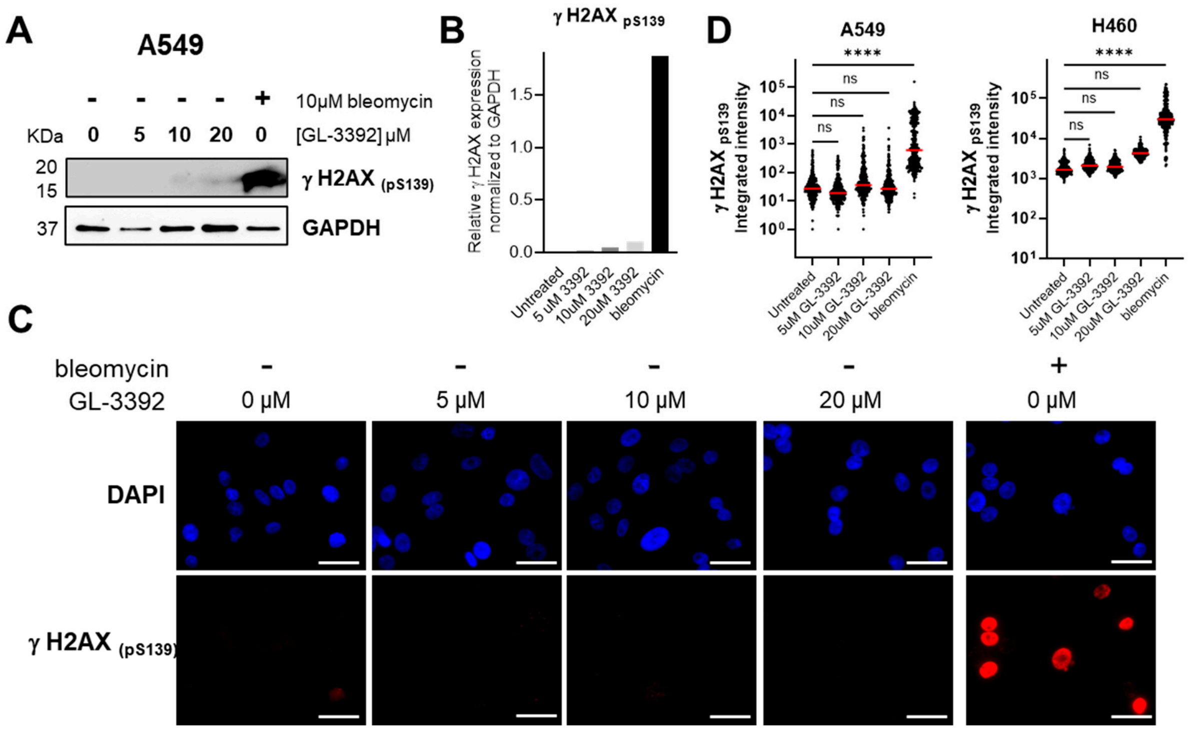

Supplementary Figure S6. GL-3392 single agent effect on DNA damage in NSCLC. (A) Western blot analysis from A549 cells nuclear extracts. (B) Densitometric analysis of bands from western blot immunodetection. The original membranes can be found in File S1 (Appendix for Supplementary Figure S6A). (C) γH2AX expression levels determined by Immunofluorescence. IF staining of DAPI nuclear staining (blue) and γH2AX (pS139) (red). Scale bars: 10□m. (D) Quantitation of integrated fluorescence intensity of γH2AX foci represented as the median of 300 nuclei per field. ns: not significant, *****p* <0.0001 as calculated by one-way ANOVA with Dunnett's multiple comparisons test.
