# Supplementary material for: Impact of Optimized Ku–DNA Binding Inhibitors on the Cellular and In Vivo DNA Damage Response
Source: Cancers (Basel). 2024 Sep 26;16(19):3286. doi: 10.3390/cancers16193286 (PMC11475570; doi:10.3390/cancers16193286)
Supplement: Supplementary file 1 [file cancers-16-03286-s001.zip › File S1. The original Western blot membranes.pdf]

Appendix for Figure 6B

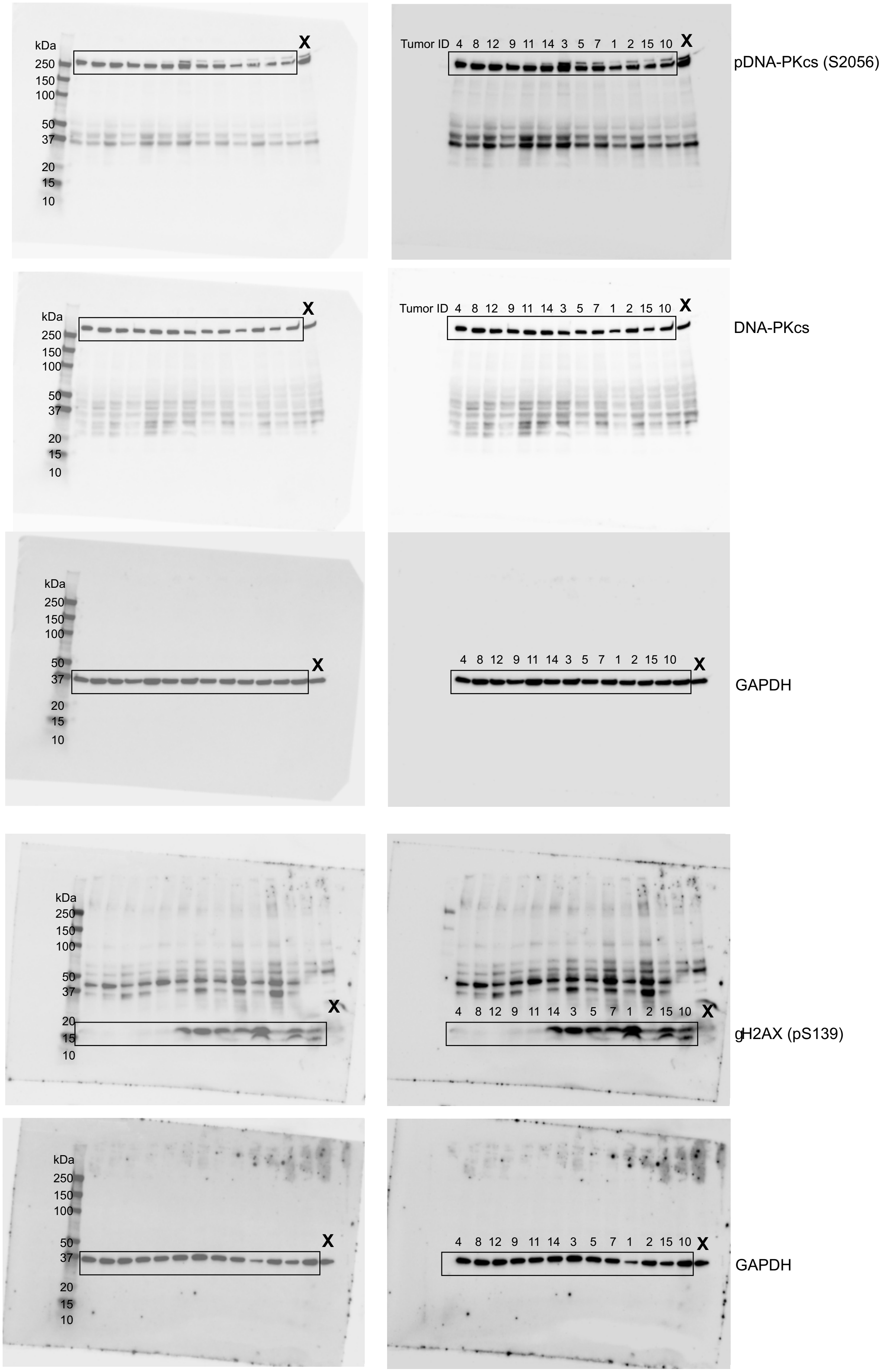

Appendix for Figure 6F

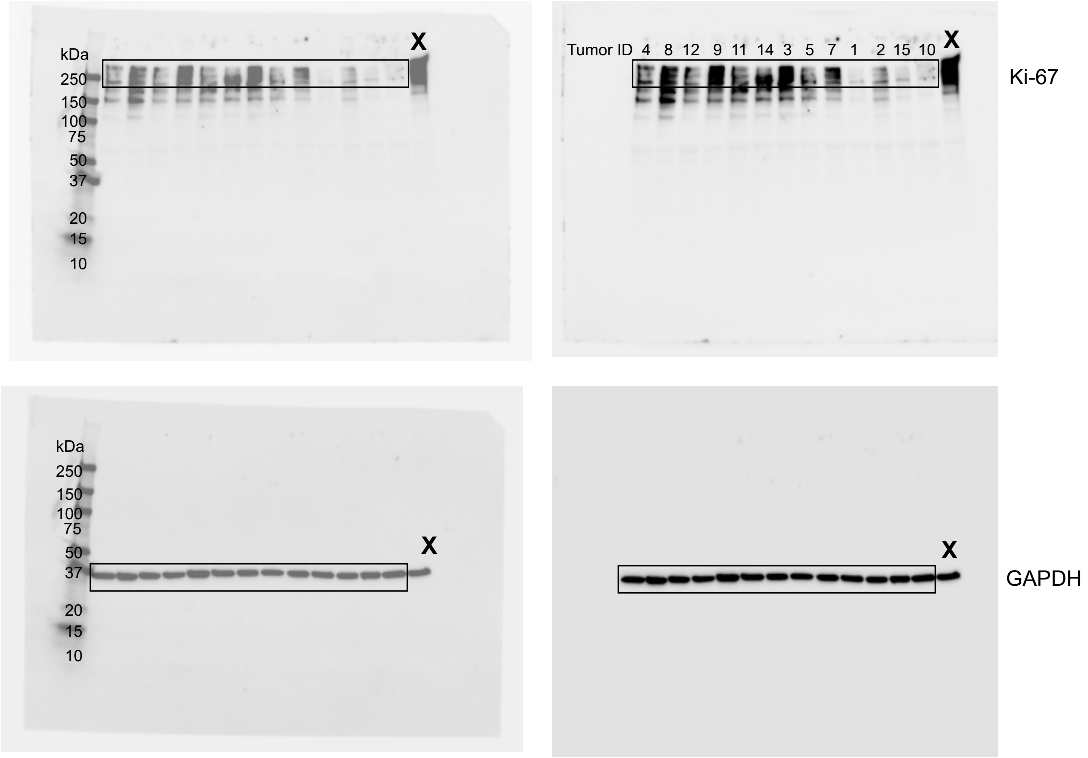

Appendix for Supplementary Figure S6A

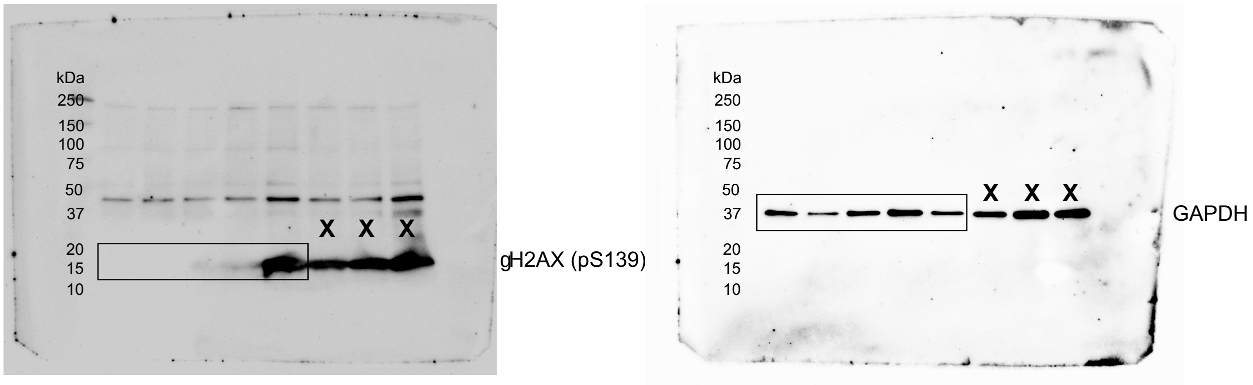

File S1. The original Western blot membranes.
